# Use of biomedical photonics in gynecological surgery: a uterine transplantation model

**DOI:** 10.4155/fsoa-2017-0129

**Published:** 2018-02-06

**Authors:** Srdjan Saso, Neil T Clancy, Benjamin P Jones, Timothy Bracewell-Milnes, Maya Al-Memar, Eleanor M Cannon, Simran Ahluwalia, Joseph Yazbek, Meen-Yau Thum, Tom Bourne, Daniel S Elson, James Richard Smith, Sadaf Ghaem-Maghami

**Affiliations:** 1 Honorary Clinical Lecturer, Division of Surgery & Cancer, Institute of Reproductive & Developmental Biology, Imperial College London, Hammersmith Hospital Campus, Du Cane Road, London, UK; 2 Junior Research Fellow, Hamlyn Center for Robotic Surgery, Institute of Global Health Innovation, Imperial College London, UK; 3 Department of Surgery & Cancer, Imperial College London, UK; 4 Clinical Research Fellow, Department of Surgery & Cancer, Institute of Reproductive & Developmental Biology, Imperial College London, Hammersmith Hospital Campus, Du Cane Road, London, UK; 5 Clinical Research Fellow, Department of Obstetrics & Gynaecology, Chelsea & Westminster Hospital, Imperial College London, UK; 6 Consultant Gynaecological Oncologist, West London Gynaecological Cancer Center, Queen Charlotte's Hospital, Hammersmith Hospital Campus, Imperial College London, Du Cane Road, London, UK; 7 Fertility Specialist, The Lister Hospital, Chelsea, London, UK; 8 Adjunct Professor in the Department of Surgery & Cancer, Faculty of Medicine, Division of Surgery & Cancer, Institute of Reproductive & Developmental Biology, Imperial College London, Hammersmith Hospital Campus, Du Cane Road, London, UK; 9 Professor of Surgical Imaging & Biophotonics, Hamlyn Center for Robotic Surgery, Institute of Global Health Innovation, Imperial College London, London, UK; 10 Consultant Gynaecological Surgeon, West London Gynaecological Cancer Center, Queen Charlotte's Hospital, Hammersmith Hospital Campus, Imperial College London, Du Cane Road, London, UK; 11 Academic Reader, Division of Surgery & Cancer, Institute of Reproductive & Developmental Biology, Imperial College London, Hammersmith Hospital Campus, Du Cane Road, London, UK

**Keywords:** optical imaging, oxygen saturation, surgical imaging, tissue perfusion, uterine transplantation

## Abstract

**Aim:**

Uterine transplantation (UTx) has been proposed as a treatment for permanent absolute uterine factor infertility. The study aims were to compare pulse oximetry and multispectral imaging (MSI), for intraoperative tracking of uterine oxygen saturation in animal UTx models (rabbit and sheep).

**Results/methodology:**

Imaging results confirmed the re-establishment of adequate perfusion in the transplanted organ after surgery. Comparison of oxygen saturation* *values between the pre-UTx donor and post-UTx recipient, and pre-UTx and post-UTx recipient reveals a statistically significant decrease in saturation levels post-UTx.

**Conclusion:**

The use of MSI is the first case in gynecology and has demonstrated promise of possible future human use. MSI technique has advantages over pulse oximetry – it provides spatial information in a real-time, noncontact manner.

## Lay abstract

Transplanting wombs into women has been offered as a potential solution to treat women who have had them removed previously or who were born without one. Our aim here was to compare two techniques: pulse oximetry and multispectral imaging, for checking oxygen levels in the newly transplanted womb. The experiments were performed in two types of animals, rabbit and sheep. The use of multispectral imaging is the first case in gynecology and has demonstrated promise of possible future human use. It has advantages over pulse oximetry – it provides valuable information in a real-time, non-contact manner.

In current medical practice, tissue pathology is diagnosed by the application of two different processes: volumetric tissue visualization and tissue biopsy. The information provided does not overlap, and a missing link becomes apparent: the absence of real-time, functional and molecular data, pre-, intra- and post-operatively. Absence of data at a molecular level causes serious pathology (e.g., cancer, graft rejection) not to be diagnosed when ‘abnormal’ change is occurring at that level, which first precedes and subsequently triggers anatomic alterations.

Biomedical photonics is a field with potential to bridge the gap between the macroscopic and microscopic methodologies described above. It studies the interaction between light and tissue, and can provide information on processes occurring at a molecular level, both at microscopic spatial and nanometer spectral resolutions [1,2]. Today, biomedical photonics is a well-established and commonly utilized tool in endoscopy, laser therapy and microscopy.

Uterine transplantation (UTx) is a surgical procedure proposed for the treatment of absolute uterine factor infertility, whereby the absence of the uterus renders a woman unconditionally infertile. The feasibility of UTx to offer the potential for pregnancy to women diagnosed with absolute uterine factor infertility has been demonstrated by the first live birth following UTx. The biggest challenge is posed by potential ischemia and reperfusion injury. The critical stage of this operation is the uterus reperfusion in the recipient, following anastomosis of graft to donor vessels during which ischemic injury can cause parenchyma and microcirculatory impairment [3]. A need therefore exists for a technique which will be able to assess the level of ischemia and reperfusion injury, and provide related diagnostic information during and post-UTx, as well as prior to possible pregnancy.

Biomedical photonics has the potential to do just that by processing data related to hemoglobin concentration and oxygen saturation (O_2_Sat). Importantly, the data will be noninvasive, real time, localized and high resolution. A type of photonics known as reflectance spectroscopy has been successfully used to diagnose chronic mesenteric ischemia during endoscopy [4,5].

## Aim

This study assessed whether two photonic applications, pulse oximetry (PO) and multispectral imaging (MSI), could be used to assess the level of ischemia and reperfusion injury in two animal UTx models.

## Materials & methods

The theory behind biomedical photonics is summarized in Appendix A of the Supplementary Document. Full details of the surgical procedures and parameters, ischemic time and pathological findings may be found in separate papers relating to the rabbit [6] and sheep transplants [7]. All animal experiments described in this paper were conducted under UK Home Office licenses (70/7508 and 70/6927).

### Pulse oximetry

PO is a noninvasive method of measuring the arterial O_2_Sat of hemoglobin. It has been applied previously by this team to assess uterine perfusion and viability immediately following pelvic surgery, including UTx [8,9]. A probe is placed around the cornua which is linked to a microprocessor unit displaying a waveform, O_2_Sat and pulse rate. Two light-emitting diodes are contained within the probe. The light they emit passes through the cornua to a photodetector. Some of the light is absorbed by blood and soft tissues during its passage through a select tissue. The amount of absorption in general and at each light wavelength is proportional to the concentration of hemoglobin within the tissues. The microprocessor can then calculate the proportion of oxygenated hemoglobin by computing the absorption at the two wavelengths. The pulse oximeter measures O_2_Sat and perfusion index (PI). PI is independent of O_2_Sat and acts as an indicator of total blood volume. The oximeter produces a graph related to the amount of light absorbed by the tissue over time. The microprocessor can select out the absorbance of the pulsatile fraction of blood (arterial flow) from absorbance of nonpulsatile venous or capillary blood and other tissue pigments.

**Figure 1.**
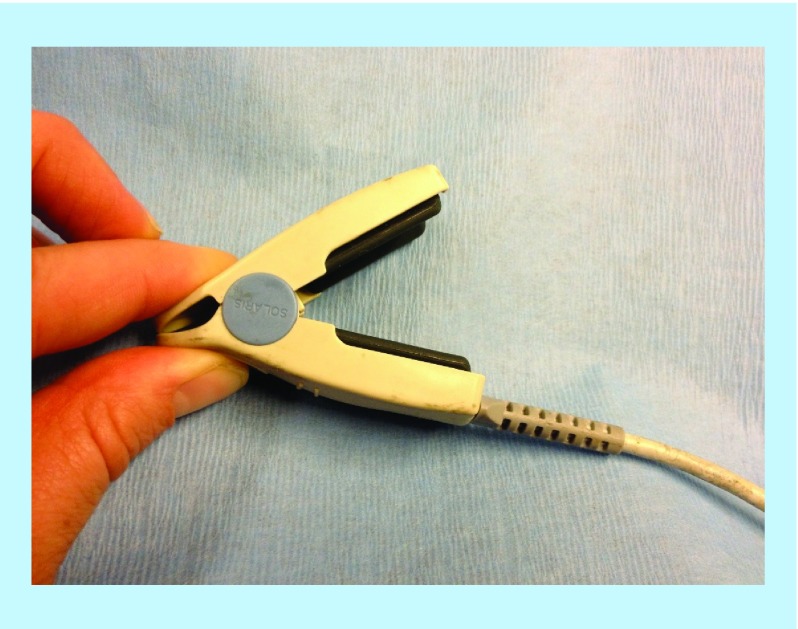
**Modified finger probe to allow wider opening of the jaws to fit satisfactorily around the uterine cornua.**

In this experiment, a normal finger PO probe was modified to allow the jaws to open wider so as to fit around the uterine horns (Figure 1). It was placed in a sterile endoscopic bag/drape to maintain aseptic technique. Once in direct contact with the uterine tissue, it measured O_2_Sat and PI, two independent variables [10,11]. This was possible as the oximeter (Datex-Ohmeda 3600P, KY, USA) has the capacity to measure both variables. The heart rate recorded on the uterine oximeter was constantly compared with that of the anesthetic oximeter to ensure accuracy. The probe was placed on both the medial and lateral aspects of the right and left uterine horns. This area of the organ is relatively thin allowing infrared light needed in oximetry to pass through the structure. Use of a superficial marker suture on the broad ligament allowed for consistency in the probe site application.

The timing of each measurement was standardized. Donor and recipient native uteri were scanned immediately following laparotomy, with the recipient donor uterus scanned just prior to closure. In the allogeneic rabbit model, we modified the application in comparison to its previous use by applying it on three separate occasions: prior to graft retrieval from donor – donor uterus; prior to recipient hysterectomy – recipient native uterus; and post-transplantation – recipient donor uterus. In the first two instances, readings were taken once normal pelvic anatomy had been established and uterine vasculature was skeletonized. Readings were taken post-UTx following visual confirmation of uterine viability by restoration of its pink pretransplant appearance.

In the autogeneic sheep model, measurements were taken on two separate occasions: prior to graft retrieval, and 30-min post UTx.

PI is a relative indicator of blood volume at the probe site. PI measurements were used to compare volume in a longitudinal fashion in the same animal because blood volume at the probe site is relative to individual patient tissue and vessel elasticity. Therefore, the age, weight, stage of the menstrual cycle or any anatomic variants of the rabbit does not interfere with the comparisons. Blood flow studies in a longitudinal manner have been successfully utilized previously in the description of variation of blood flow in the testis [12] and ovary [13].

### Minimally invasive optical spectroscopy

MSI requires a multispectral laparoscope to acquire high-resolution images of changes in tissue O_2_Sat. The technique has been applied in current medical practice in a number of ways: gingival inflammation quantification [14], brain tumor demarcation [15], fundus analysis [16], imaging of Hirschsprung's disease [17] and analysis of facial skin lesions [18]. It has yet to be applied in gynecology. This technique involves using a white light source and tuneable filter to acquire images at many wavelengths in the visible range in order to build up a reflectance spectrum at each pixel [19]. Here, like with PO, hemoglobin concentration and O_2_Sat, as well as scattering decide the shape of the visible light spectrum reflected from tissue. These spectra are then fitted using a linear regression model which uses prior knowledge of the optical absorption spectra of oxy- and deoxy-hemoglobin, from which their relative contributions are calculated. Previous implementations of this technique have also used hyperspectral data with numerical techniques to aid tissue visualization and ischemia detection [19,20]. MSI refers to techniques that use a relatively low number of wavelengths (up to 40–50) while hyperspectral imaging refers to techniques that can apply hundreds of wavelengths. A strict threshold between the two types does not exist but ‘hyper’ usually means hundred or more.

**Figure 2.**
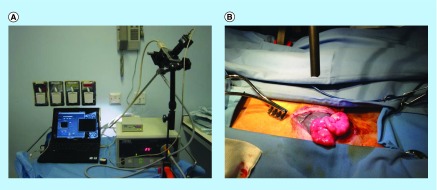
**Multispectral imaging laparoscope.** **(A)** Multispectral imaging laparoscope with Xenon light source and control computer; **(B)** multispectral imaging laparoscope in action over the operative field.

**Figure 3.**
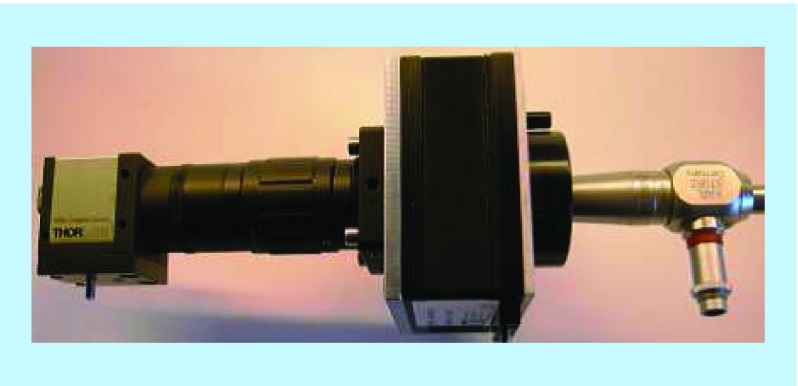
**Hyperspectral laparoscopic system consisting in a charged-coupled device camera and a liquid crystal tuneable filter attached to a regular rigid laparoscope.**

The laparoscope (Figures 2A & B & 3) consisted of a Liquid Crystal Tuneable Filter (VariSpec, Cambridge Research & Instrumentation, Inc., MA, USA) that transmitted light with an average bandwidth of 15 nm as well as a computer-controlled central wavelength between 400 and 720 nm. The light emitted by the Xenon light source (model 20133020, Karl Storz GmbH, Tuttlingen, Germany) ranged between 400 and 700 nm. It was then subsequently transmitted by a fiber bundle light guide and coupled into the 12 mm rigid 30 degree laparoscope (Storz GmbH). The sample was usually placed 50 mm from the distal tip of the laparoscope. The laparoscope was secured to a flexible mounting arm and the whole system placed on a trolley that could be conveniently positioned at the bedside for measurements. The distance between the laparoscope tip and the organ was 10 cm and the field-of-view of the system is 6 cm in diameter (Figure 2B).

To form an image on a monochrome-charged-coupled device (CCD) camera (DCU223M, Thorlabs, Inc., NJ, USA), an additional 50 mm focal length lens was inserted at the proximal end. This lens was then mounted in a helicoid barrel to adjust the image plan to the laparoscope-sample distance. The lens was placed between the LCTF and the CCD with the laparoscope attached to the LCTF via a clip. This allowed easy removal of the LCTF/CCD block [21]. To generate reflected intensity spectra of the tissue at each spatial location, a data cube of 13 images (500–620 nm) was acquired. Exposure times of approximately 200 ms/image were required, resulting in a total acquisition time of approximately 3.2 s. In order to compensate for misalignments caused by breathing and peristalsis motion during this time, a preprocessing image registration step using custom-written feature-tracking software was carried out [22,23].

The spectral resolution is best described as the bandwidth of the LCTF transmission spectra. It was evaluated every 10 nm, from 500 to 620 nm, by recording the light reflected by the Xenon lamp from a spectralon reflectance standard (Labsphere, Inc., NH, USA), with a spectrometer (HR4000, Ocean Optics, Inc., NH, USA) through the hyperspectral laparoscope (minus the camera). The key element of MSI is a tuneable filter. Its band-pass filter, a device capable of passing wavelengths within a certain range and rejecting wavelengths outside that range, can be electronically tuned. This allows a high degree of flexibility in the choice of wavelengths to be used, and the order in which they are acquired, compared with filter wheel technologies.

Both the rabbit and sheep uteri were imaged with the left and right cornua, and fallopian tubes visible. In the rabbit, this was performed prior to transplantation – donor uterus; prior to recipient hysterectomy – recipient native uterus; and post-transplantation – recipient donor uterus. In the sheep, measurements were taken prior to retrieval and 30 min following commencement of reperfusion (i.e., post-UTx).

Relative concentrations of oxygenated and deoxygenated hemoglobin were determined using linear least squares regression [21] of the experimental data to the known pure component spectra [24]. The sum of the concentrations (total hemoglobin) was calculated, along with the concentration of oxygenated hemoglobin expressed as a percentage of total hemoglobin (O_2_Sat). All data processing was conducted offline using MATLAB (The Math Works, MA, Inc.) [21].

### Data presentation & analysis

The data have been presented using 2D graphs, 2D images, histopathology slides, figures and descriptive tables. Data related to O_2_Sat and PI were defined as nonparametric, and Mann–Whitney U test was therefore carried out for analysis. A statistically significant difference was applied for a p-value < 0.05. All statistical analysis was done using the Statistical Package for the Social Sciences version 19 (SPSS, Inc., IL, USA).

## Results

Each transplant procedure is referred to by a UTx number (rabbits: 1–9; sheep: 1–5) corresponding to the results described in our surgical papers [6,7]. The resolution achieved with the MSI laparoscope was approximately 0.3 mm/pixel at a working distance of 10 cm.

### Rabbit model

**Figure 4.**
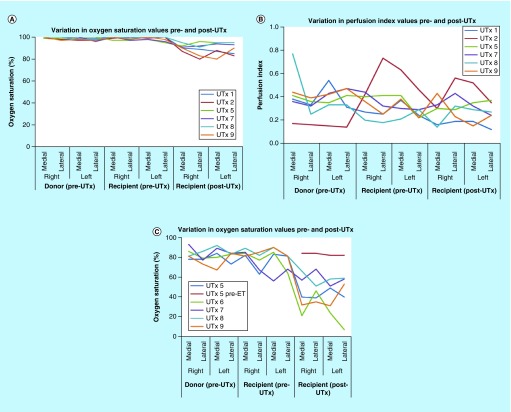
**Variation in oxygen saturation and perfusion index values when measured with a pulse oximeter and a multispectral imaging laparoscope (rabbit model).** **(A)** Oxygen saturation values of the donor and the recipient rabbit measured with a pulse oximeter; **(B)** Perfusion index values of the donor and the recipient rabbit measured with a pulse oximeter **(C)**; Oxygen saturation values of the donor and the recipient rabbit measured with a multispectral imaging laparoscope. ET: Embryo transfer; UTx: Uterine transplantation.

PO was applied in all nine transplantations. As the does died intraoperatively in UTx #3, #4 and #6, no values for O_2_Sat and PI were obtained on the recipient donor uteri (post-UTx). The averages and standard deviations for these measurements are provided in Tables 1 and 2. Comparison of O_2_Sat values between the pre-UTx donor and post-UTx recipient, and pre-UTx recipient and post-UTx recipient revealed a statistically significant decrease in saturation levels post-UTx. A similar comparison of PI values did not demonstrate any fall in PI level post-UTx, and importantly no statistical difference. This variation in O_2_Sat and PI values is depicted in Figure 4A–C.

**Table 1.** **Oxygen saturation values of the donor and the recipient rabbit (measured with a pulse oximeter).**

**Rabbit**	**Side**	**Cornua**	**Oxygen saturation (%)**									**Mean ± SD (%)**

			**UTx1**	**UTx 2**	**UTx 3**	**UTx 4**	**UTx 5**	**UTx 6**	**UTx 7**	**UTx 8**	**UTx 9**	**All UTx**
Donor (pre-UTx)	Right	Medial	99	99	93	98	99	96	100	100	100	98 ± 0.02

		Lateral	98	98	95	99	99	99	100	100	97	98 ± 0.02

	Left	Medial	99	97	96	100	100	97	100	98	98	98 ± 0.02

		Lateral	99	97	87	96	100	98	96	98	97	96 ± 0.04

Recipient (pre-UTx)	Right	Medial	100	99	98	99	97	100	100	100	100	99 ± 0.01

		Lateral	100	100	96	98	97	97	97	99	98	98 ± 0.01

	Left	Medial	100	99	92	97	98	100	98	100	100	98 ± 0.03

		Lateral	100	100	99	99	95	92	96	100	98	98 ± 0.03

Recipient (post-UTx)	Right	Medial	90	87	NA	NA	92	NA	91	95	90	91 ± 0.03

		Lateral	89	80	NA	NA	96	NA	92	91	83	89 ± 0.06

	Left	Medial	87	88	NA	NA	95	NA	94	95	80	90 ± 0.06

		Lateral	85	83	NA	NA	95	NA	93	95	90	90 ± 0.05

NA: Non applicable; SD: Standard deviation; UTx: Uterine transplantation.

**Table 2.** **Perfusion index values of the donor and the recipient rabbit measured with a pulse oximeter.**

**Rabbit**	**Side**	**Cornua**	**Perfusion index**									**Mean ± SD**

			**UTx 1**	**UTx 2**	**UTx 3**	**UTx 4**	**UTx 5**	**UTx 6**	**UTx 7**	**UTx 8**	**UTx 9**	**All UTx**
Donor (pre-UTx)	Right	Medial	0.38	0.17	0.44	0.41	0.41	0.19	0.36	0.77	0.44	0.40 ± 0.17

		Lateral	0.33	0.16	0.15	0.36	0.36	0.13	0.32	0.25	0.39	0.27 ± 0.10

	Left	Medial	0.54	0.15	0.15	0.35	0.35	0.38	0.43	0.33	0.42	0.34 ± 0.13

		Lateral	0.31	0.14	0.25	0.41	0.41	0.28	0.47	0.33	0.47	0.34 ± 0.11

Recipient (pre-UTx)	Right	Medial	0.27	0.42	0.25	0.40	0.40	0.59	0.44	0.20	0.36	0.37 ± 0.12

		Lateral	0.25	0.73	0.13	0.41	0.41	0.43	0.32	0.18	0.25	0.35 ± 0.18

	Left	Medial	0.37	0.63	0.35	0.41	0.41	0.56	0.30	0.21	0.38	0.40 ± 0.13

		Lateral	0.24	0.46	0.23	0.22	0.22	0.17	0.29	0.29	0.22	0.26 ± 0.08

Recipient (post-UTx)	Right	Medial	0.16	0.30	NA	NA	0.30	NA	0.33	0.14	0.43	0.28 ± 0.11

		Lateral	0.19	0.56	NA	NA	0.29	NA	0.43	0.32	0.23	0.34 ± 0.14

	Left	Medial	0.19	0.52	NA	NA	0.35	NA	0.32	0.29	0.15	0.30 ± 0.13

		Lateral	0.12	0.35	NA	NA	0.37	NA	0.24	0.27	0.24	0.27 ± 0.09

NA: Non applicable; SD: Standard deviation; UTx: Uterine transplantation.

Optical spectroscopy was applied in UTx #3–9. The averages and standard deviations for O_2_Sat are provided in Table 3. Images of the donor uterus, recipient native uterus and recipient donor uterus were obtained in all seven transplants. Following the reanastomosis of the inferior vena cava (IVC) and abdominal aorta (AA), the uterus was observed to undergo a color shift during reperfusion from its blanched appearance after flushing to a more reddish color. However, as the does died intraoperatively in UTx #3 and #4, O_2_Sat measurements were calculated with an MSI laparoscope only on the recipient donor uteri #5–9 (post-UTx). Even though recipients #6 and #8 also died intraoperatively, measurements were obtained in these cases as deaths occurred 18 and 23 min after the donor uterus had been transplanted and reperfusion had been established. Baseline O_2_Sat levels in both the donors’ and recipients’ native uteri are high in all the rabbit models, varying from 87 to 100% (mean: 98%). For a particular organ, the transplanted uterus shows a consistently lower O_2_Sat immediately post-UTx than the native graft across the entire dataset. This is also borne out by the pulse oximeter readings for UTx #5, #7–9. The MSI results are lower than the pulse oximeter in every case. Recipient #5 was the only doe to survive long term and underwent an embryo transfer process to see if pregnancy could be achieved following allogeneic UTx. As a result, an additional measurement was taken on day 89 postoperatively just prior to the embryo transfer. Mean O_2_Sat in the graft recovered to 86% from the immediate postoperative value of 47%, which is also higher than the original baseline reading of 80% (medial aspect).

**Table 3.** **Oxygen saturation values of the donor and the recipient rabbit measured with a multispectral imaging laparoscope.**

**Rabbit**	**Side**	**Cornua**	**O_2_ saturation (%)**						**Mean ± SD (%)**

			**UTx 5**	**UTx 5 pre-ET**	**UTx 6**	**UTx 7**	**UTx 8**	**UTx 9**	**All UTx**
Donor (pre-UTx)	Right	Medial	83	NA	76	86	93	81	80 ± 5

		Lateral	89	NA	92	79	77	86	91 ± 2.1

	Left	Medial	90	NA	88	80	89	92	89 ± 1.4

		Lateral	82	NA	86	83	84	83	84 ± 2.8

Recipient (pre-UTx)	Right	Medial	89	NA	83	84	85	89	86 ± 4.2

		Lateral	89	NA	88	77	67	82	89 ± 0.7

	Left	Medial	93	NA	88	85	56	90	91 ± 3.5

		Lateral	78	NA	81	64	68	81	80 ± 2.1

Recipient (post-UTx)	Right	Medial	60	84	44	21	57	66	55 ± 0.21

		Lateral	58	84	51	46	68	51	60 ± 0.14

	Left	Medial	59	82	68	24	51	58	57 ± 0.19

		Lateral	63	82	93	7	58	59	60 ± 0.30

ET: Embryo transfer; NA: Non applicable; SD: Standard deviation; UTx: Uterine transplantation.

**Figure 5.**
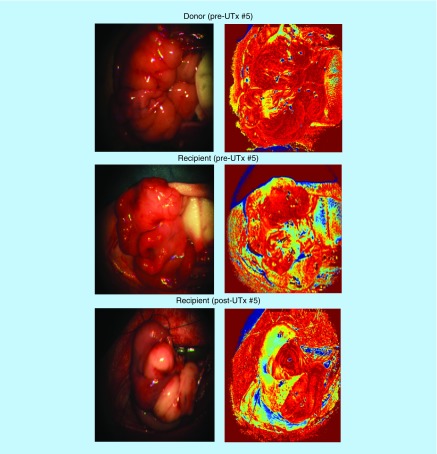
**Uterine transplantation #5.** The images on the left are standard images of the rabbit cornua produced using a red green blue filter. The images on the right are reference tissue O_2_Sat images. The O_2_Sat images are displayed using a color scale that ranges from ‘0’ (dark blue) to ‘1’ (bright red). UTx: Uterine transplantation.

**Figure 6.**
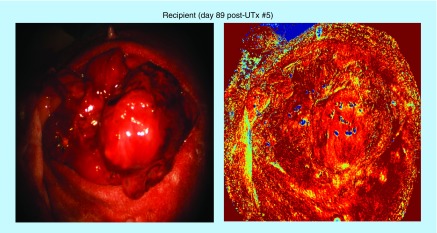
**Images taken on day 89 post uterine transplantation (#5) just prior to embryo transfer.** UTx: Uterine transplantation.

The processed O_2_Sat images for the donor uterus pre- and post-UTx, along with color images reconstructed from the multispectral data after integration under the red green blue (RGB) filter transmission response of a digital color camera are shown in Figures 5 and 6 (UTx #5 used as example). The pre-UTx images show that the graft is well perfused in the donor doe, with high oxygen content in both cornua. The area of low O_2_Sat in the center may correspond to a less vascularized area or possibly an area with a higher venous to arterial supply ratio. The post-UTx images show similar features after blood supply is re-established from the AA. Areas of low O_2_Sat correspond to connective tissue where reperfusion is expected to last the longest. Here, the comparison of O_2_Sat values between the pre-UTx donor and post-UTx recipient, and pre-UTx recipient and post-UTx recipient revealed a statistically nonsignificant decrease in saturation levels post-UTx. Figure 6 indicates the changes that the graft has undergone in the intervening period. A significant number of postoperative adhesions have hidden the uterus's distinctive shape. Despite this, it is clear that perfusion has been fully re-established.

### Sheep model

**Figure 7.**
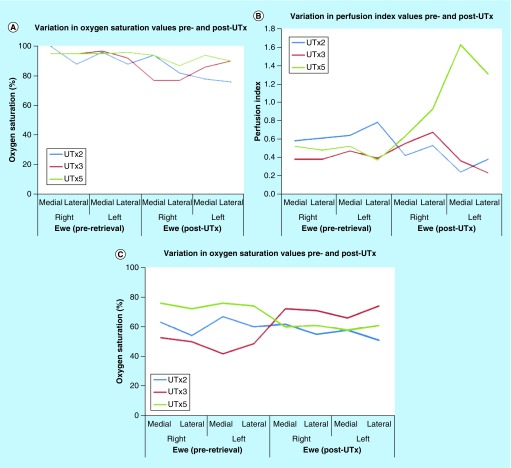
**Variation in oxygen saturation and perfusion index values when measured with a pulse oximeter and a multispectral imaging laparoscope (sheep model).** **(A)** Oxygen saturation values pre- and post-autotransplant measured with a pulse oximeter; **(B)** Perfusion index values pre- and post-autotransplant measured with a pulse oximeter; **(C)** Oxygen saturation values pre- and post-autotransplant measured with a multispectral imaging laparoscope. UTx: Uterine transplantation.

PO was applied in all five autotransplants. As the uterus was not retrieved in UTx #1 and the ewe died intraoperatively in UTx #4, no values for O_2_Sat and PI were obtained post-UTx. The averages and standard deviations for these measurements are provided in Table 4. Comparison of O_2_Sat values prior to graft retrieval and post-UTx revealed a statistically significant decrease in saturation levels post-UTx. A similar comparison of PI values did not demonstrate any fall in PI level post-UTx, and importantly no statistical difference. This variation in O_2_Sat and PI values is depicted in Figure 7A and B.

**Table 4.** **Oxygen saturation and perfusion index values when measured with a pulse oximeter (sheep model).**

**Sheep**	**Side**	**Cornua**	**O_2_ saturation (%)**					**Mean ± SD (%)**	**Perfusion index**					**Mean ± SD**

			**UTx 1**	**UTx 2**	**UTx 3**	**UTx 4**	**UTx 5**	**All UTx**	**UTx 1**	**UTx 2**	**UTx 3**	**UTx 4**	**UTx 5**	**All UTx**
Ewe (pre-retrieval)	Right	Medial	94	100	95	96	95	96 ± 0.02	0.34	0.58	0.38	1.25	0.52	0.61 ± 0.37

		Lateral	92	88	95	95	95	93 ± 0.03	0.44	0.61	0.38	1.33	0.48	0.65 ± 0.39

	Left	Medial	94	96	97	94	95	95 ± 0.01	0.23	0.64	0.47	1.55	0.52	0.68 ± 0.51

		Lateral	96	88	92	96	96	94 ± 0.04	0.55	0.78	0.39	1.50	0.37	0.72 ± 0.47

Ewe (post-UTx)	Right	Medial	NA	94	77	NA	94	88 ± 0.10	NA	0.42	0.55	NA	0.63	0.53 ± 0.11

		Lateral	NA	82	77	NA	87	82 ± 0.05	NA	0.53	0.67	NA	0.93	0.71 ± 0.20

	Left	Medial	NA	78	86	NA	94	86 ± 0.08	NA	0.24	0.36	NA	1.63	0.74 ± 0.77

		Lateral	NA	76	90	NA	90	85 ± 0.08	NA	0.38	0.23	NA	1.31	0.64 ± 0.59

NA: Non applicable; SD: Standard deviation; UTx: Uterine transplantation.

Optical spectroscopy was applied for all five transplants. However, for reasons explained above, images of the uterus prior to retrieval and post-UTx were obtained only in UTx #2, #3 and #5. Following the anastomosis of the internal to external iliac vessels [6], the uterus was observed to undergo a color shift during reperfusion from its blanched appearance after flushing to a more reddish color. The averages and standard deviations for O_2_Sat are provided in Table 5, with variation depicted in Figure 7C.

**Table 5.** **Oxygen saturation values when measured with a multispectral imaging laparoscope (sheep model).**

**Sheep**	**Side**	**Cornua**	**O_2_ saturation (%)**					**Mean ± SD (%)**

			**UTx 1**	**UTx 2**	**UTx 3**	**UTx 4**	**UTx 5**	**All UTx**
Ewe (pre-retrieval)	Right	Medial	76	63	53	74	76	68 ± 0.10

		Lateral	70	54	50	73	72	64 ± 0.11

	Left	Medial	65	67	42	64	76	63 ± 0.13

		Lateral	70	60	49	69	74	64 ± 0.10

Ewe (post-UTx)	Right	Medial	NA	62	72	NA	60	65 ± 0.06

		Lateral	NA	55	71	NA	61	62 ± 0.08

	Left	Medial	NA	58	66	NA	58	61 ± 0.05

		Lateral	NA	51	74	NA	61	62 ± 0.12

NA: Non applicable; SD: Standard deviation; UTx: Uterine transplantation.

**Figure 8.**
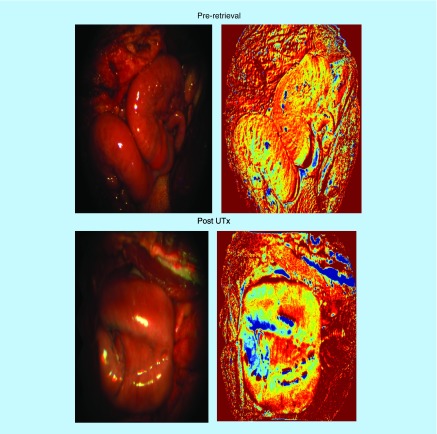
**Images correspond to uterine transplantation #5.** The images on the left are standard images of the sheep cornua produced using a red green blue filter. The images on the right are reference tissue oxygen saturation images. The oxygen saturation images are displayed using a color scale that ranges from ‘0’ (dark blue) to ‘1’ (bright red). UTx: Uterine transplantation.

The processed O_2_Sat images of the uterus prior to retrieval and post-UTx, along with color images reconstructed from the multispectral data after integration under the RGB filter transmission response of a digital color camera are shown in Figure 8. The pre-retrieval images show that the graft appears bright red/pink, is well perfused in the donor doe, with high oxygen content in both cornua. The area of low O_2_Sat in the center may correspond to a less vascularized area or possibly an area with a higher venous to arterial supply ratio. The post-UTx images show similar features after blood supply is re-established from the external iliac arteries. The transplanted uterus (‘graft’), while also red, is not as saturated as in the native state. The processed MSI data also support this, with the bright red and yellow shades indicating relatively high oxygenation in both cases. Areas of low O_2_Sat correspond to connective tissue where reperfusion is expected to last the longest. Here, the comparison of O_2_Sat values between the uterus prior to retrieval and post-UTx revealed a statistically nonsignificant decrease in saturation levels post-UTx.

## Discussion

The ultimate aim of UTx is to enable a feasible allogeneic transplant with respect to anatomy and vascular viability, in order to allow for embryo transfer and pregnancy at a later date. The visual appearance of the graft, O_2_Sat and PI measurements and optical spectrometry were used to ensure adequate graft reperfusion immediately after UTx.

### Pulse oximetry & perfusion index

Two previous studies used PO to investigate the vasculature of the infundibulopelvic and broad ligaments, assess the contribution of the ovarian and uterine vessels to overall uterine perfusion, and consider the clinical applications of selective pelvic vessel ligation [25,26]. Conclusions drawn implied that uterine and ovarian vessels contribute almost equally to uterine perfusion. Furthermore, an O_2_Sat level of 40–50% maintained by only two supplying arteries, appear to be perfectly adequate to ensure a healthy and viable uterus.

In rabbit UTx #1, #2, #5, #7–9, where the recipient did not die intraoperatively, O_2_Sat measurements taken after graft perfusion were statistically different to the pretransplant values of both donor and native uteri. The appliance was sensitive enough to detect a statistically significant 10% reduction in O_2_Sat pre- and post-UTx. Promisingly, however, the mean absolute O_2_Sat values were still 89–90%. This is a level perfectly adequate to maintain a viable uterus. PI was statistically not different before retrieval and after completion of the transplantation. During an abdominal radical trachelectomy procedure, the ovarian arteries are the only two arteries that are maintained as the uterine blood supply. Despite this, a woman can still have a normal menstrual cycle as well as fall pregnant following this operation.

The sheep cohort produced results which confirmed the above findings. Only three out of five sheep received the autograft (UTx #2, #3 and #5), and from those three the O_2_Sat post-UTx ranged from 82 to 88%. PI was again statistically not different before retrieval and after completion of the transplantation. This means that in the lager animal model, where technically the team had a much easier access to the vessels, and a larger surgical field to work on, uterine perfusion, as well uterine blood flow were adequately high at the end of the surgery.

The difference in the pattern of O_2_Sat and PI values pre- and post-UTx can be explained by two factors. The anastomosis appears to be patent in the immediate postoperative period which would explain the consistency in PI value. PI is an indicator of blood volume as explained previously. However, the delay in vessel vasodilatation secondary to both cold and warm ischemia, as well as a minimal reperfusion time between reanastomosis and PO would have contributed to a reduction in O_2_Sat.

The limitations of this model are self evident. Positioning and manipulating the probe and cornua is a practically difficult task. The oximeter requires constant contact with the target in question, that is, cornua, can be ‘trial and error’ at times, and is calibrated on human volunteers. It provides a point measurement of arterial oxygenation giving no spatial information on the overall supply to the tissue. Finally, probe size and positioning must be correct, with excessive pressure avoided at all times to prevent cornual damage. The number of separate factors which can affect data derived from PO calls for an improved and more precise tool.

### Optical spectroscopy

On comparison of its methodology with PO, an MSI laparoscope appears to have some advantages. First, the technique to monitor cornual O_2_Sat over the entire visible section of the organ is fast and non contact. Furthermore, one does not have to ‘wrestle’ with the cornua to find a strong pulsatile signal. All these might be important for stages of the operation where time is critical.

In comparison to PO in the rabbit model, measured values of both cornual O_2_Sat show a statistically significant but greater decrease after transplantation. The absolute values of O_2_Sat are therefore much lower, on average approximately 25%. This is lower than the decrease recorded with a pulse oximeter. This may reflect the fact that pulse oximeters measure arterial O_2_Sat only, while this imaging method also takes the venous side into account in addition to areas of the tissue that are not well perfused, reducing the mean value. This also explains the lower oxygenated area in the center. With regards to the most important part of the grafting process, reanastomosis of IVC and AA, the O_2_Sat map demonstrated a successful anastomosis as highlighted by the O_2_Sat regions close to the region where AA was reconnected.

MSI was performed postoperatively only once and on one rabbit recipient only. This was the only rabbit that had survived long term, with the measurement taken on the day of the embryo transfer (day 89). Interestingly, the level of perfusion was almost back to preoperative levels when measured on day 89 post-UTx (83 versus 85%, respectively). It is difficult to draw conclusions based on just one result but it should be highlighted that a pregnancy was achieved with that specific uterine graft. In addition, it seems that small vessel neovascularization of the uterus occurs once the abdomen is closed, which restores the uterine oxygen saturation to preoperative levels (or from 58% in the immediate postoperative period to 83% on day 89).

The results demonstrated with the sheep model are extremely promising. There was no statistically significant difference between the pre-retrieval and post-UTx O_2_Sat values, which ranged 63–68% respectively. A perfusion level above 60% would be more than acceptable to ensure future fertility, especially as we know that the level is likely to increase in the long term, secondary to uterine vascular neogeneration. The results are reassuring as the sheep model closely resembles the female pelvis, and the type of vessel anastomosis (internal to external iliac vessels) applied will be identical in a human model.

One of the major disadvantages that arise with optical spectroscopy is motion artifacts. They are introduced by breathing, peristalsis and relaxation of tissue during acquisition of the image stack, with peristalsis of cornua the major issue in our model. Motion artifacts were corrected using a registration algorithm based on ‘sparse feature correspondences’, whose effectiveness has been demonstrated for motion tracking of a beating heart endoscopically [27].

We strongly believe that MSI will be of use in human UTx in the future. When assessing the overall picture, hyperspectral imaging may be an efficient tool to assess uterine tissue reoxygenation in both time and space in the period immediately following the reanastomosis, as well as in the postoperative period overall. The aim is to extend the use and potential benefit of MSI to gynecological surgery. In particular, fertility-sparing surgery where one would be curious to know the perfusion and, hence, viability of an organ following removal of a tumor contained within that tissue. A good example would be removal of a borderline ovarian tumor within an ovary, with ovarian tissue left behind after to ensure fertility. A further use could be during colposcopy to see whether MSI could reveal cervical intra-epithelial neoplasia (CIN)/cancerous areas of the cervix.

## Conclusion

Mapping of O_2_Sat is now possible over the entire uterine graft and visible range, as opposed to a few selected points. The use of MSI in this scenario is the first such case with respect to gynecological surgery and has demonstrated promise of possible future use in a human model.

## Future perspective

MSI has the potential to become a very effective tool in transplant surgery secondary to its ability to give a real-time picture of graft perfusion and therefore graft viability. The ability to obtain such results will allow the surgical team to act appropriately and most importantly intraoperatively in order to rectify any issues.

The next goal therefore is to evaluate the effect of hypo-oxygenation on the long-term organ function. We will evaluate uterine viability with various functional long-term aspects to confirm the utility of MSI.

**Executive summary**

- Uterine transplantation (UTx) has been proposed as a treatment for permanent absolute uterine factor infertility.
- Uterine reperfusion with oxygenated blood following anastomosis of the vessels during UTx is necessary to ensure uterine survival.
- The study aims were to explore and compare two optical measurement techniques, pulse oximetry and multispectral imaging (MSI), for intraoperative tracking of uterine oxygen saturation in animal UTx models.
- The use of MSI is the first such case in gynecology and has demonstrated promise of possible future use in humans.
- MSI technique has advantages over pulse oximetry – it can provide spatial information in a real-time, non-contact manner.
