# Supplementary material for: Use of biomedical photonics in gynecological surgery: a uterine transplantation model
Source: Future Sci OA. 2018 Feb 6;4(4):FSO286. doi: 10.4155/fsoa-2017-0129 (PMC5905613; doi:10.4155/fsoa-2017-0129)
Supplement: Supplementary file 1 [file fsoa-04-286-s1.docx]

**Appendix A**

The manner in which light interacts with tissue (absorption, transmission, diffuse reflection, fluorescence) may vary, but it carries the common goal of supplying more complete tissue diagnostic information to the surgeon with minimal trauma as well as procedure duration. To date, the technique has shown to be advantageous over medical imaging and biopsy in a number of cases. First, it is atraumatic to the patient, whereas ionising radiation is used in medical imaging and biopsy involves tissue cutting and therefore damage. Second, biomedical photonics can generate immediate data at the time of an operation which therefore speeds up diagnosis and subsequent management. With medical imaging and biopsy one is forced to wait for a period of time until the data is processed. Third, the resolution of biomedical photonics is highly detailed, with the ability to characterize tissue in the micrometric range and thus, probe biochemical alterations that pre-stage pathology. This is definitely not the case in medical imaging and biopsy, where a clear demarcation line between diseased and healthy tissue is often unclear.

The principle of biomedical photonics is based on the complex phenomenon that light is modified by tissue, with the spectral range of interest lying in the non-ionizing and richly diagnostic visible domain (400nm - 700 nm). When striking the surface of a tissue, light can either be reflected or refracted. Reflected light is of no particular interest as it does not penetrate the tissue and therefore does not contain significant diagnostic information. It is referred to as a specular reflection. The amount of refracted and reflected light depends on the refractive index at the air/tissue interface and the angle between the in-coming light beam and the tissue normal. The photons forming the refracted light can be absorbed or scattered by the tissue chromophores and scatterers. The fraction of the photons which emerge from the tissue after multiple scattering events is called diffuse reflectance as the scattering induces the spreading and the loss of directionality of the incident beam.

**Assessing oxygen saturation using pulse oximetry**

A probe is placed around the cornua which is linked to a microprocessor unit displaying a waveform, O_2_Sat and pulse rate. Two light emitting diodes are contained within the probe. The light they emit passes through the cornua to a photodetector. Some of the light is absorbed by blood and soft tissues during its passage through a select tissue. The amount of absorption in general and at each light wavelength is proportional to the concentration of haemoglobin within the tissues. The microprocessor can then calculate the proportion of oxygenated haemoglobin by computing the absorption at the two wavelengths. The pulse oximeter measures O_2_Sat and perfusion index (PI). PI is independent of O_2_Sat and acts as an indicator of total blood volume. The oximeter produces a graph related to the amount of light absorbed by the tissue over time. The microprocessor can select out the absorbance of the pulsatile fraction of blood (arterial flow) from absorbance of non-pulsatile venous or capillary blood and other tissue pigments.

**Forming an image using Multi Spectral Imaging**

The technique has been applied in current medical practice in a number of ways: gingival inflammation quantification,^14^ brain tumour demarcation,^15^ fundus analysis,^16^ imaging of Hirschsprung’s disease,^17^ and analysis of facial skin lesions.^18^ This technique involves using a white light source and tuneable filter to acquire images at many wavelengths in the visible range in order to build up a reflectance spectrum at each pixel.[^19^](#_ENREF_177)^,20^

To form an image on a monochrome charged-coupled device (CCD) camera (DCU223M, Thorlabs, Inc., USA), an additional 50mm focal length lens was inserted at the proximal end. This lens was then mounted in a helicoid barrel to adjust the image plan to the laparoscope-sample distance. The lens was placed between the LCTF and the CCD with the laparoscope attached to the LCTF via a clip. This allowed easy removal of the LCTF/CCD block ^21^. To generate reflected intensity spectra of the tissue at each spatial location, a data cube of 13 images (500 to 620 nm) was acquired. Exposure times of ∼200 ms∕image were required, resulting in a total acquisition time of ∼3.2 s. In order to compensate for misalignments caused by breathing and peristalsis motion during this time, a preprocessing image registration step using custom-written feature-tracking software was carried out ^22,23^.

The spectral resolution is best described as the bandwidth of the LCTF transmission spectra. It was evaluated every 10 nm, from 500nm to 620nm, by recording the light reflected by the Xenon lamp from a spectralon reflectance standard (Labsphere, Inc., USA), with a spectrometer (HR4000, Ocean Optics, Inc., USA) through the hyperspectral laparoscope (minus the camera). The key element of multispectral imaging is a tuneable filter. Its band-pass filter, a device capable of passing wavelengths within a certain range and rejecting wavelnegths outside that range, can be electronically tuned. This allows a high degree of flexibility in the choice of wavelengths to be used, and the order in which they are acquired, compared to filter wheel technologies.

Relative concentrations of oxygenated and deoxygenated haemoglobin were determined using linear least squares regression ^21^ of the experimental data to the known pure component spectra.^22-24^ The sum of the concentrations (total haemoglobin) was calculated, along with the concentration of oxygenated haemoglobin expressed as a percentage of total haemoglobin (oxygen saturation; StO2). All data processing was conducted offline using MATLAB (The Math Works, Inc.).^21^

***Data Presentation and Analysis***

The data has been presented using 2D graphs, 2D images, histopathology slides, figures and descriptive tables. Data related to O_2_Sat and PI was defined as non-parametric and *Mann-Whitney U test* was therefore carried out for analysis. A statistically significant difference was applied for a p-value <0.05. All statistical analysis was done using the Statistical Package for the Social Sciences version 19 (SPSS Inc, Chicago, Illinois, USA).
